# Grand Challenges in Nutrition

**DOI:** 10.3389/fnut.2014.00001

**Published:** 2014-03-20

**Authors:** Johannes le Coutre

**Affiliations:** ^1^Nestle Research Center, Lausanne, Switzerland

**Keywords:** nutrition, food, eating behavior, obesity, malnutrition, metabolism, food supply, hunger

No subject pertains more to human life than nutrition. Biologically, this statement is evident. Historically, we are aware of the impact nutrition can have on entire cultures as a result of abundant or diminished and impaired or restricted food supply. Nutrition and food always have been at the heart of human life.

Owing to the significance of food and nutrition, the world is facing a continued set of challenges and *Frontiers in Nutrition* is determined to address all of them. Specifically, in an ever more densely populated world, we will continue to be confronted by problems of hunger on a massive scale, hence *Eradicating Extreme Poverty and Hunger* is the number one Millenium Development Goal of the UN (1) and the key issue for the post-2015 development agenda (2).

At the same time, we observe a growing obesity epidemic in both developed countries and in emerging economies at an alarming velocity.

Hunger, malnutrition, and obesity are linked to a variety of health and societal issues, such as impaired development, diabetes, Alzheimer’s disease, and allergies, as well as an environmental burden and impaired economic performance – to name just a few.

Yet, at the beginning of the twenty-first century, we are also embracing an era full of opportunity with breathtaking advances in science, agriculture, commerce, and global interaction.

So, how do these challenges in the field of nutrition translate into tangible challenges for *Frontiers in Nutrition*? Ultimately, *Frontiers’* ambition has to be directed toward the alleviation of hunger, malnutrition, and obesity. With a sober mindset this can be achieved through the publication of credible, rigorous, and meaningful science within the specialty sections of the journal.

## More Science is Needed

Over the past decades, general awareness about the biological aspects of nutrition has grown. The over-simplistic view that nutrition refers mainly to the supply of macro- and micro-nutrients through carbohydrates, fat, protein, and a mix of vitamins and minerals to provide building blocks and energy has been replaced by novel approaches that aim to introduce a substantially more detailed level of understanding. Today, we are more conscious of the complex nature of the changing demands of our bodies in different environments and situations and, in particular, as we grow older (3).

In addition, mounting scientific evidence indicates that certain foods can help treat diseases and are already playing a strong role in the prevention of diseases (4–6). In recent years, research on nutrition and nutrient requirements has taken increasingly into account personalized and genetic information with the goal to cater directly to individual needs either based on different genotypes or on different metabolic states of the body.

However, more needs to be done and complex scientific questions need to be answered. In the overall context of nutrition, an opportunity and an equal need is emerging for ground breaking and integrated fundamental science on nutrition physiology, brain mechanisms, overall health, childhood development, and nutrition methodology (7, 8).

For example, in the specific case of weight management even a few fundamental questions can already provide ample work for years to come: What are the mechanisms of food perception and how can the molecular mechanisms of hunger and satiety homeostasis in our brain be integrated with food intake behavior? What can we learn from the population dynamics of about 2 kg of intestinal microbiota to formulate tangible advice for healthy nutrition? (4).

## Nutrition in Society, Private, and Public Sector

Nutrition is involved with culture, infrastructure, and economic status and as such it is a prerequisite for and a result of our lifestyle at the same time. The world is changing and so will the actual diets, and the food related behaviors change – for better or worse.

Centuries ago, adequate nutrition was a question of appropriate food supply within well-defined geographies and populations. We have come a long way from being hunters and gatherers to the formation of today’s complex societies driving agriculture, local food manufacturing, and a globally acting food industry. With the exponential growth of an aging world population and society’s desire to fight malnutrition and climate change, new demands arise for the sourcing and distribution of food.

So, where does our food come from and who is responsible for it? For example, the spice trade likely accounts for the first documented long-distance transport of food ingredients across the globe. But is it still sustainable today to ship food around the world? And how do recommended dietary guidelines impact the carbon footprint on our ecosystems?

With the key objective of feeding the planet, it is crucial to establish a meaningful and forward-looking framework among universities, the private sector, regulatory bodies, and NGOs in order to ensure adequate nutrition for all – a topic that is too important to be handled behind closed doors.

## Frontiers in Nutrition

In the globalized world of the twenty-first century transparence and communication are the key determinants behind driving change. And change is needed. As scientific funding is becoming scarcer, increased scrutiny on the “meaningfulness” of scientific research and publication will become the dominant factor. *Frontiers in Nutrition* will address the need for science that is asking the right questions in a vast and complex network of open issues around nutrition.

The challenge will be to illustrate scientific results in a new light, to integrate these results with interdisciplinary problems and to conclude on actions that will have an impact on the global agenda.
